# Interfacing DNA with Gold Nanoparticles for Heavy Metal Detection

**DOI:** 10.3390/bios10110167

**Published:** 2020-11-06

**Authors:** Zhiyu He, Huiling Yin, Chia-Chen Chang, Guoqing Wang, Xingguo Liang

**Affiliations:** 1 College of Food Science and Engineering, Ocean University of China, Qingdao 266003, China; hezhiyu@stu.ouc.edu.cn (Z.H.); yinhuiling@stu.ouc.edu.cn (H.Y.); liangxg@ouc.edu.cn (X.L.); 2 Laboratory for Marine Drugs and Bioproducts, Pilot National Laboratory for Marine Science and Technology (Qingdao), Qingdao 266237, China; 3 Department of Medical Biotechnology and Laboratory Sciences, College of Medicine, Chang Gung University, Taoyuan 33302, Taiwan; chang@mail.cgu.edu.tw; 4 Kidney Research Center, Department of Nephrology, Chang Gung Memorial Hospital, Taoyuan 33302, Taiwan

**Keywords:** biosensing, DNA, gold nanoparticles, interface, heavy metal

## Abstract

The contamination of heavy metals (e.g., Hg, Pb, Cd and As) poses great risks to the environment and human health. Rapid and simple detection of heavy metals of considerable toxicity in low concentration levels is an important task in biological and environmental analysis. Among the many convenient detection methods for heavy metals, DNA-inspired gold nanoparticles (DNA-AuNPs) have become a well-established approach, in which assembly/disassembly of AuNPs is used for colorimetric signaling of the recognition event between DNA and target heavy metals at the AuNP interface. This review focuses on the recent efforts of employing DNA to manipulate the interfacial properties of AuNPs, as well as the major advances in the colorimetric detection of heavy metals. Beginning with the introduction of the fundamental aspects of DNA and AuNPs, three main strategies of constructing DNA-AuNPs with DNA binding-responsive interface are discussed, namely, crosslinking, electrostatic interaction and base pair stacking. Then, recent achievements in colorimetric biosensing of heavy metals based on manipulation of the interface of DNA-AuNPs are surveyed and compared. Finally, perspectives on challenges and opportunities for future research in this field are provided.

## 1. Introduction

Environment and food contamination by heavy metals and the resulting adverse effects to the ecosystem and human health have been issues of increasing concern worldwide [1]. Particularly, mercury (Hg), lead (Pb), cadmium (Cd) and arsenic (As) are widespread toxic pollutants [2], among which arsenic as a metalloid is often classified as a heavy metal in terms of its similar contamination sources and molecular mechanisms of toxicity as compared to other heavy metals [3]. It is worth noting that all of the four heavy metals appear in the top 10 chemicals of major public health listed by the World Health Organization (WHO) [4]. By forming stable complexes with essential proteins or enzymes, the heavy metals can interfere with their biological functions in metabolism and antioxidant protection, thereby inducing toxicological and carcinogenic effects on tissues and organs [1,5,6]. Since each of the heavy metals, even in small quantities, may cause potent human health effects that depend on the heavy metal’s oxidation state and solubility [7,8], many countries and international organizations have accordingly defined maximum allowable levels of heavy metals in food, drinking water and other mediums [9]. On the other hand, simple and sensitive testing of heavy metals has become important in environmental analysis, food analysis and medical diagnosis [10].

The innovation of biosensing tools boosted by the development of bionanotechnology provides new avenues to convenient heavy metal detection. As an emerging class of recognition elements, functional DNAs, such as aptamer and DNAzyme, have been screened by combinational biotechnological process, where magnetic beads are often employed to extract and enrich DNA-target complexes magnetically [11,12,13]. Aptamers are known as DNA or RNA sequences that can specifically bind with molecules or proteins, while DNAzymes are a class of DNA capable of catalyzing reactions (e.g., RNA cleavage); in many cases, using metal ions as cofactors [14]. Both functional DNAs offer high selectivity to the target, and have been utilized for the detection of toxic heavy metals in optical [15] and electrochemical [6] biosensing. On the other hand, gold nanoparticles (AuNPs), which exhibit localized surface plasmon resonance (LSPR), represent an advantageous signal transducer for recognition behaviors over other indicators [16], such as dyes [17], metal oxides [18], coordination complexes [18], polymers [18,19,20] and enzymes [21], due to the high extinction coefficient, stability and biocompatibility [22,23]. AuNPs can further have their plasmon coupled when they are brought into close proximity, promising distance-dependent optical properties. Based on the combined use of functional DNAs and AuNPs, accordingly, a platform for colorimetric biosensing of heavy metals has become established [24,25]. In comparison to other functional DNA-based sensing strategies, such as electrochemical [6] and fluorescent [26] methods, colorimetric biosensing using DNA-AuNPs offers the benefits of convenience, intuitiveness and elimination of instruments, which are desirable for field analysis.

**Figure 1:**
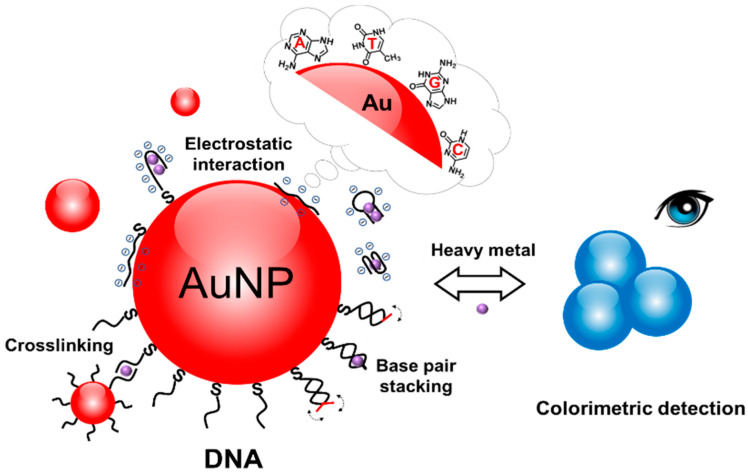
Schematic illustration of surface engineering of gold nanoparticles (AuNPs) with heavy metal-specific DNA.

The responsiveness of interfacial property of DNA-AuNPs to a target is the key to make the biosensor work [27]. The recognition events of the functional DNA to heavy metal species involved at the interface of AuNPs is the major driving force for a responsive interfacial property of DNA-AuNPs, and thereby controls the assembly of DNA-AuNPs [27,28]. It has been accepted that, by binding with heavy metal species, aptamers and DNAzymes undergo changes in their confirmation and base pairing status [29]. These events often greatly alter the interfacial interactions among the DNA-AuNPs, thereby triggering the assembly/disassembly of the DNA-AuNPs for signifying the target heavy metal species (Figure 1). The fine design of the characteristic parameters of DNA-AuNPs, including the DNA sequences and the morphology of AuNPs, is the core content of building such DNA-AuNP colorimetric biosensors. 

This review focuses on the major advances in heavy metal detection based on DNA-AuNPs, with an emphasis on how DNA integrate with AuNPs to form a sensing platform. This paper starts with a brief introduction of the fundamental aspects of DNA and AuNPs; the mechanisms of DNA recognition-induced interfacial assembly of AuNPs based on crosslinking, electrostatic interaction or base pair stacking will be classified and discussed. Then, methods for colorimetric detection of the widespread toxic heavy metals based on the above mechanisms are surveyed in detail, including Hg, Pb, Cd and As. Finally, conclusions on the recent progress and perspectives for future research trends are made, to provide useful information for researchers who work in related fields and to address the challenges for DNA-AuNPs to be applied in detecting a wide range of heavy metals.

## 2. The Mechanism of DNA-AuNPs’ Interfacial Assembly for Heavy Metal Detection

The conformational change caused by the interaction with heavy metals accounts for the recognition behavior of functional DNA [29]. For example, DNAzymes can be catalyzed to cleave on the deoxynucleotide site with the assistance of metal ions [30], while aptamers can be folded to a secondary structure by binding to heavy metals with specific bases [31]. In addition, thymine and cytosine can be mismatched with the same base as themselves, by forming stable T−Hg^II^−T or C-Ag^I^-C complexes [32,33]. 

For effectively manipulating the interface of colloidal AuNPs with the recognition behavior of functional DNA, the fashion of integration between DNA and AuNPs should be considered first [34,35]. In particular, the sequence, length, label and concentration of DNA are key factors that can affect the density and conformation of DNA on a given AuNP. Additionally, there is no doubt that the charge, curvature and surfactant of the AuNP surface play essential roles in determining the manner of integration between DNA and AuNPs [36,37,38]. Although the many characteristics of DNA and AuNPs make their interactions complicated, researchers did develop reliable methods to characterize and control them [39,40,41,42,43]. 

### 2.1. Fundamental Aspects of AuNPs and DNA

AuNPs are nanostructured gold consisting of at least thousands of atoms. Dispersed in aqueous solution, AuNPs are in general isolated from each other. The interparticle distance is nearly a thousand of nanometers, which is much larger than their diameter. The strong LSPR effect makes their plasmon band fall in visible regions, appearing in a brilliant reddish or bluish color in a colloidal state that is dependent on their morphologies [23,44]. Spherical AuNPs synthesized by citrate-assisted thermal reduction of HAuCl_4_ are among the mostly adopted nanostructures [45]. The capping agent determines the surface charge of the AuNPs to be negative [42]. For gold nanorods and nanoplates prepared using cetyltrimethylammonium (CTA) halide [46,47], their surfaces are positively charged. Furthermore, their surface activity is site-dependent due to the structural anisotropy, which has implications in region-selective modification and deposition of species of interest [48,49,50,51]. 

The structural fundamentals endow DNA withcomplex chemical functionalities, and thus are the cause of the smart characteristics of DNA molecules discovered in both biological and non-biological science [52]. Owing to the linear structure consisting of four types of nucleobases (A, G, C, T) that are linked by sugar-phosphate backbone, as well as the ease of chemical modification, DNA can integrate with gold surfaces via physicochemical adsorption or covalent binding. 

#### 2.1.1. Adsorption of DNA onto AuNPs

Although the negatively charged AuNPs capped by citrate are repulsive to the polyanionic DNA backbones, attraction interactions exist and enable the adsorption of unmodified DNA oligonucleotides onto negatively charged AuNPs [53]. Related research has shown that DNA bases have stable coordination interactions with AuNPs via ring nitrogen, exocyclic amino or keto groups in the purine or pyrimidine rings [41], with the binding strength dependent on their chemical structure [54,55]. Adenine possesses the highest binding affinity to AuNPs [56]. It binds with the surface of AuNPs via the exocyclic amino group and the N7 atom [41]. Secondary to adenine, guanine and cytosine interact with AuNPs via the keto group and the neighboring nitrogen [55]. Thymine shows the weakest adsorption to the negatively charged AuNPs, with a possible interaction site through a keto oxygen atom only [41]. The adsorption and desorption of DNA on the surface of AuNP can be controlled by the conformational change of DNA, altering the surface charge of AuNPs. 

#### 2.1.2. Covalent Binding

Thiol-tagged DNA can bind with AuNPs covalently through the formation of Au-S bound. This innovative configuration of DNA-AuNPs was pioneered by Mirkin et al. Alivisatos et al. have enabled a great deal of applications [57,58]. With the single-point attachment on AuNPs, DNA molecules protrude into solution, providing more opportunities for engineering the interface of DNA-AuNPs in a three-dimensional space for colorimetric determination of heavy metals. When DNA is grafted on AuNPs in a relatively low density, a variety of recognition events are facilitated, including DNA hybridization and target binding [59]. Based on improved methods for surface DNA modification, such as salt-aging [39,60], lowering pH [61,62] and freezing [62,63], the surface of AuNP can be further encapsulated by a radiative and dense single-stranded (ss) DNA monolayer. The high density of DNA brings great electrostatic repulsion and steric hindrance to the particle surfaces, endowing AuNPs with a good colloidal stability, even in a high-salt environment [22]. In this case, the entropic repulsion reveals its control over the assembly of AuNPs, which is relevant to the mobility of DNA that grafted on AuNPs.

### 2.2. Heavy Metal-Responsive Interface of DNA-AuNPs

Once DNA is integrated with AuNPs, the design of the interface among DNA-AuNPs will be crucial for a sensitive response to target heavy metals [64]. For the controlled interfacial assembly of DNA-AuNPs, connecting neighboring DNA-AuNPs by a heavy metal-enabled DNA base pairing has been one of the well-recognized strategies [57]. Accompanied with a red-to-purple color shift, the interparticle distance among the crosslinked DNA-AuNPs may be predicted or simulated based on the design of the DNA sequence [65,66]. This method is thought to be considerably sensitive, since in principle just one ssDNA crosslinker can connect two DNA-AuNPs and sufficiently shorten the interparticle distance [67].

The dispersion of DNA-AuNPs in a colloidal solution usually relies on the electrostatic repulsion among them [68]. The charge distribution of DNA is an essential factor that may influence the colloidal stability of DNA-AuNPs, which may be altered by heavy metal binding. This electrostatics-mediated assembly of DNA-AuNPs for heavy metal detection can be based on either adsorbed [53,69] DNA or covalently grafted [70] DNA on AuNPs.

If it has a high salt concentration, the surface charge of grafted DNA can be screened [71]. The entropy of DNA-AuNPs that is proportional to the flexibility of the grafted DNA becomes decisive in driving the interfacial assembly of DNA-AuNPs. For instance, the entropic repulsion among AuNPs grafted with dense double-strand (ds) DNA can be controlled through terminal DNA base pairing, which was discovered by Maeda and coworkers [72]. When the outermost DNA base pairs are mismatched, the AuNPs remain dispersed even at high ionic strength, due to the increased entropic repulsion caused by the fraying motion of unpaired bases [73]. When the dsDNA is fully matched, however, base pairing can restrict the flexibility of the DNA terminals and lead to hydrophobic interaction among the DNA-AuNPs [74,75], thereby inducing the assembly of the dsDNA-AuNPs. It has been established that the status of a single base pairing controlled by the heavy metal-mediated mismatch can cause assembly/disassembly of the dsDNA-AuNPs, which suggest a good potential sensitive colorimetric detection [76,77].

## 3. Applications of DNA-AuNPs to Heavy Metal Detection

In a sensor based on colloidal DNA-AuNP, AuNP is the color indicator, whereas surface DNA molecules are responsible for the recognition of a heavy metal target. The interface of DNA-AuNPs represents a wide space for the binding events. Depending on the design of the sensor, the binding between DNA and the target can either crosslink DNA-AuNPs or trigger great change in the interfacial charge and entropic properties of DNA-AuNPs. The binding-induced interfacial assembly/disassembly of DNA-AuNPs for the sensing of heavy metals will be discussed.

### 3.1. Target-Driven Crosslinking of DNA-AuNPs

**Figure 2:**
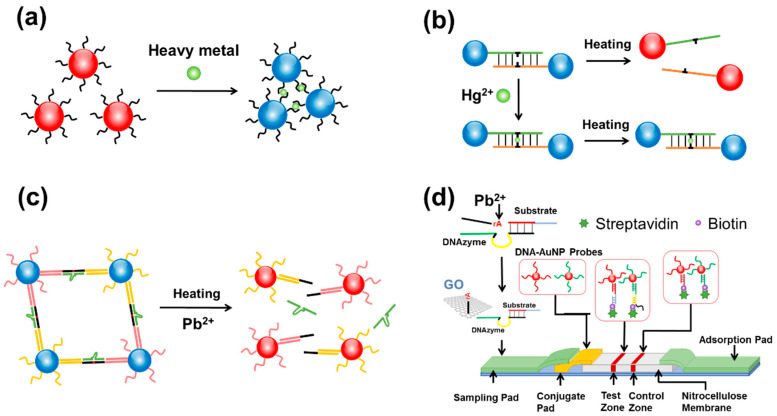
Crosslinking assembly of DNA-AuNPs for heavy metal detection. (**a**) Heavy metal as the crosslinker for the DNA-AuNP assembly. (**b**) Hg^2+^ detection based on the melting temperature of DNA-AuNP assemblies that are regulated by the formation of T−Hg^II^−T duplexes. (**c**) Pb^2+^ detection based on the DNA-AuNPs network that are crosslinked by DNAzymes. (**d**) Schematic representation of the strip biosensor for Pb^2+^ detection based on GR-5 DNAzyme and AuNPs.

Among the various strategies for colorimetric detection of analytes using DNA-AuNPs, crosslinking assembly of DNA-AuNPs induced by target binding is the most straightforward (Figure 2a) [78]. In a crosslinking-based detection scheme, neighboring DNA–AuNPs are brought into close proximity via target binding, which thereby form sandwich configurations that induce a color shift from red to blue. Over the past two decades, this methodology has been developed for the detection of various Hg species and Pb^2+^ at low concentration levels [79].

Due to Hg^2+^-mediated base pairing of T−T mismatch, two functional ligands that both involve T may also be connected in the presence of Hg^2+^ [33,80]. It has also been reported that CH_3_Hg^+^ binds with T in a stronger manner [81], which is transformed from Hg^2+^ by microbial processing in nature and can be accumulated through aquatic food chains with dramatically increased pathogenicity [82]. Aulsebrook et al. prepared AuNP probes that were modified with a T moiety via covalent bound between Au and dithiol group, which can chelate both Hg^2+^ and CH_3_Hg^+^ [83]. The ligand consists of a T group linked to lipoic acid by a polyethylene glycol spacer, which helps enhance the negative charge of the AuNP probes by the lipoic acid group and thereby increases the colloidal stability through electrostatic repulsion. Upon incubation of Hg^2+^ or CH_3_Hg^+^ at 40 °C for 5 min, the AuNP probes undergo assembly in a crosslinking manner accompanied with a color shift from red to blue. Due to the induced different hues of blue, Hg^2+^ and CH_3_Hg^+^ could also be distinguished in the assay. Nevertheless, minimal spectral change could be observed for Hg^2+^ at concentrations lower than 20 μM, and a limit of detection (LOD) of ~15 μM was obtained.

In the above detection scheme, AuNPs were crosslinked by formation of the complex of Hg species and a T-based moiety anchored on AuNPs. Lee et al. further developed a selective and sensitive colorimetric method for Hg^2+^ detection, which relied on the use of two types of DNA-AuNPs that show complementary sequences, except for an intentional T−T mismatch (Figure 2b) [84]. In this design, Hg^2+^ could complex with the T−T mismatch and help form a tightly crosslinked assembly that have higher melting temperatures. In the absence of Hg^2+^, crosslinked AuNPs appeared purple, which appeared red when melted at ~46 °C. By measuring the melting temperature of the DNA-AuNPs in the presence of Hg^2+^ at different concentrations, a linear detection range over 100−2000 nM of Hg^2+^ was observed, with the LOD determined to be ~100 nM for the system. Xue et al. further improved the detection system for Hg^2+^ [85]. They prepared three DNA-probes, among which one of the DNA-probes acted as a bridge between the other two DNA-probes anchored on AuNPs. By controlling the number of T−T mismatches in the complex formed by the three probes, the melting point of the complex can be suppressed below the ambient temperature. Hg^2+^ strengthens the complex and improved the melting temperature over 40 °C, distinguishing Hg^2+^ from other metal ions that have little impact on the melting points of the system. Similar to the complex of T−Hg^II^−T, C−Ag^I^−C can be formed using an Ag^+^ and C−C mismatch, which is useful for designing a colorimetric sensor for Ag^+^ [86]. Chen et al. also constructed a visual biosensor using two types of DNA-AuNPs that can be bridged by Ag^+^, based on C−Ag^I^−C formation for the detection of Ag^+^ [87,88].

In addition to binding with metals, some DNA sequences have been selected in vitro to catalyze the cleavage of phosphodiester bond in RNA, namely DNAzyme. DNAzyme-based colorimetric biosensing of Pb^2+^ has also been developed. Liu and Lu reported a method for Pb^2+^ detection using DNAzyme-linked DNA-AuNP networks, which was formed by hybridization among DNAzyme, the corresponding substrate chain and DNA-AuNP in a “head-to-tail” manner (Figure 2c) [89]. The Pb^2+^ detection range of 100 nM to 4 µM was obtained. In 2004, Liu and Lu future reported a faster detection procedure that could be operated at ambient temperature [90]. In that work, AuNPs with larger size (42 nm) were arranged in a “tail-to-tail” manner in the assembly, which responded rapidly (<10 min) to the presence of Pb^2+^ in comparison to the previous AuNP probes (2 h), although the detection limit (400 nM) for Pb^2+^ was not as low as that in the previous approach.

In order to realize fast and convenient detection in the field, colorimetric detection based on DNA-AuNPs strip has recently become a research hotspot. Wang and co-workers established a Pb^2+^ biosensor using GR-5 DNAzyme with the help of graphene oxide (GO) [91]. On the paper strip, two types of DNA-AuNP probes that were complementary to the green part of the DNAzyme and the red part of the substrate, respectively, were embedded in the conjugate pad. By linking streptavidin anchored on the nitrocellulose membrane, biotinylated DNAs (biotin-DNA) were immobilized in the test zone (TZ) and the control zone (CZ). In the TZ, two types of biotin-DNAs were immobilized to capture the DNA-AuNP probes (Figure 2d). One biotin-DNA formed a sandwich complex with the yellow part of the DNAzyme and a DNA-AuNP, while the other complexed with the blue part of the substrate and the other DNA-AuNP. In the CZ, two types of biotin-DNA probes were designed to bind both DNA-AuNP probes. When the sample contains Pb^2+^, the black part in the DNAzyme containing rA was released due to the substrate cleavage and then intercepted by GO through a stacking interaction. The rest of the DNAzyme could pass through the conjugation pad by capillary force, complexing to the DNA-AuNP probes from the pad and the biotin-DNAs and leaving a red line on the TZ for signifying Pb^2+^. Due to the specific π-stacking interaction between the ribose ring of the DNAzyme and the carbon hexagon in GO, the DNA-AuNP-based lateral flow sensor exhibited stronger specificity for Pb^2+^, thus reducing false positive interference by removing unhybridized DNAzyme and substrate. The LOD of the sensor was as low as 0.05 nM, which is much lower than the maximum allowable levels of Pb^2+^ in drinking water set by the WHO (48 nM) and the U.S. Environmental Protection Agency (EPA) (72 nM) [92,93].

### 3.2. Electrostatic Interaction-Mediated Assembly for Heavy Metal Detection

Colloidal nanoparticles are usually dispersed through interparticle electrostatic repulsion. The alternation of electrostatic force among nanoparticles may cause a change in their dispersion status. Based on electrostatic-mediated AuNP assembly/disassembly, various biosensors for heavy metals have also been established. In these works, the binding behavior of DNA to an analyte on AuNP is used to alter the interparticle electrostatic interaction, enabling change in the dispersion state of AuNPs, and thereby the detection of the analyte.

#### 3.2.1. Detection Based on Change in Electrostatic Interaction of Unmodified AuNPs

**Figure 3:**
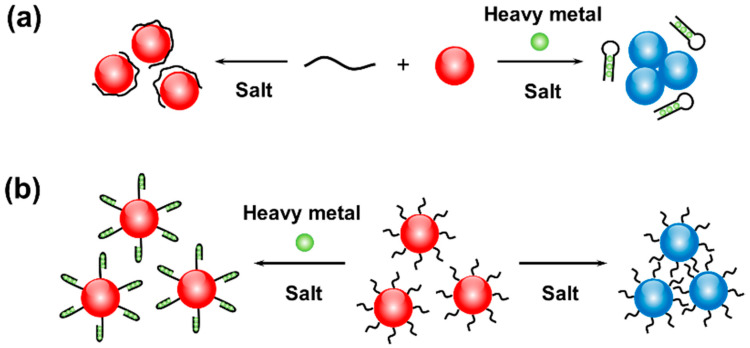
Electrostatic interaction-mediated assembly of DNA-AuNPs for heavy metal detection. (**a**) Assembly of unmodified AuNPs controlled by heavy metal binding-regulated DNA adsorption. (**b**) Assembly of DNA-grafted AuNPs controlled by heavy metal-induced DNA folding.

DNA can be adsorbed to the surface of AuNP by electrostatic and hydrophobic interaction [94]. Upon binding to a target heavy metal, DNA can undergo significant structural change. The binding behavior is often accompanied with change in the interactions among DNA-AuNPs, which further causes color change of the colloid (Figure 3a) [55].

Formation of T−Hg^II^−T by a T-rich DNA and Hg^2+^ usually induces folding of the DNA. Liu and co-workers designed a DNA-AuNP probe for detecting Hg^2+^ visually through a T−Hg^II^−T interaction [95]. In that work, they took AuNPs (13 nm diameter) and random-coil poly-Tn ssDNA in a salt solution, in which DNA adsorbed on the surface of AuNPs. Due to the high negative charge density of DNA molecules on each AuNP surface, AuNP kept dispersed state in red because of the protection by DNA. With the addition of Hg^2+^, a folded Hg^2+^-DNA complex involving T−Hg^II^−T forms, causing the complex to be desorbed from the AuNPs. Without the electrostatic repulsion provided by the adsorbed DNA, the AuNPs tend to aggregate. At a signal noise ratio of 3, the LOD was estimated to be 250 nM. Due to the C−Ag^I^−C formation by C-rich DNA and Ag^+^, a detection scheme for Ag^+^ could also be designed that is similar to the one mentioned above [96]. As a successive work to Liu’s report, Chen et al. realized field analysis of Hg^2+^ in river water with a paper-based device [97]. They first utilized an identical approach to detect Hg^2+^ in pond or polluted water samples using colloidal DNA-AuNPs, which underwent a color shift from red to blue. Next, the mixture was spotted on a paper for color signal enhancement. By cloud computing via a smartphone, the detection results for Hg^2+^ for the real samples can be readily transmitted for readout and data storage. The total test time was ~40 min with a LOD of ~50 nM for Hg^2+^-spiked pond and river water was achieved.

Toward arsenic recognition and detection, Kim et al. have screened an arsenic-bound DNA aptamer named Ars-3 in vitro, which consists of 100 nucleotides and exhibits high affinity to As^3+^ [98]. Zhan et al. designed a simple colorimetric detection method based on assembly of AuNPs, which could be regulated using the specific interaction between Ars-3 and As^3+^ [99]. Specifically, Ars-3 aptamers can be adsorbed onto the surfaces of AuNPs through base−gold interaction, which protected the AuNPs from spontaneous assembly even at high ionic strength. In the presence of As^3+^, however, the As^3+^ bound with Ars-3 and formed a complex that resulted in the base of aptamer, which was not exposed and lost the ability to adsorb on the surface of AuNPs. Consequently, the AuNPs assembled with the induction of salt with a color change from red to blue. This sensor had a detection limit of 1.26 ppb (16.8 nM) for As^3+^, which is lower than the maximum permissible concentration (10 ppb) of arsenic in drinking water determined by WHO [100].

In arsenic and its compounds, As (III) species are the most toxic and usually present in the form of arsenite. Liang and co-workers proposed a label-free colorimetric strategy to detect arsenite based on the target-induced change in the interfacial electrostatics of DNA-AuNPs [101]. The arsenite was in the form of H_3_AsO_3_, which can easily bind to the ssDNA rich in G and T, via the strong hydrogen bond [102]. The ssDNA aptamer rich in G and T and AuNPs could be integrated as the arsenite probe by adsorption. While the binding between arsenite and the aptamer altered the charge property of the aptamer, releasing the aptamer from the AuNP surfaces and inducing assembly and color change of AuNPs. The LOD was 50 ppb (396.8 nM) achieved with the naked eye and 0.5 ppb (4.0 nM) with UV/Vis spectrometry. Despite a different sequence and length, G and T-rich ssDNA sequences also function as aptamers for some other heavy metals, such as Cd^2+^. Wu et al. screened a new ssDNA aptamer named Cd-4 using the SELEX technique, which was rich in G and T and can specifically recognize Cd^2+^ [103]. The dissociation constant of Cd-4 aptamer was determined to be 34.5 nM for Cd^2+^. Subsequently, by using a Cd-4 aptamer, they tested Cd^2+^ in aqueous solution containing poly diallyl dimethyl ammonium chloride (PDDA) that can bind to Cd-4 aptamer. As a water-soluble cationic polymer, PDDA can cause the assembly of AuNPs. It binds to aptamers through non-specific electrostatic interactions. In the presence of Cd^2+^, the Cd-4 interacted strongly with Cd^2+^, resulting in the failure of binding with PDDA. Consequently, the assembly of AuNPs took place in the presence of PDDA, causing a significant change in color from wine red to blue. By using this method, Cd^2+^ can be detected at a concentration as low as 0.5 ppb (4.6 nM), which is lower than the maximum level (5 ppb) of cadmium in drinking water defined by EPA [103].

Unlike the ssDNA aptamers that fold through heavy metal binding, Pb^2+^-specific DNAzyme/substrate in a double-strand can release short nucleic acid segments for the protection of AuNPs in the presence of Pb^2+^. A blue-to-red “signal on” plasmonic sensor may be designed based on this process [104]. Taking advantage of the high specific selectivity of 8-17E DNAzyme for Pb^2+^, Wang et al. designed a label-free sensor to detect Pb^2+^ in less than 10 min [105]. In the presence of Pb^2+^, a part of the substrate chain was cut off, and this part was adsorbed on the surface of AuNPs, causing particles’ state from aggregation to dispersion. Furthermore, Memon et al. performed a colorimetric ultrasensitive detection of Pb^2+^ with DNAzyme [106]. In their design, 5-bases were removed from 8–17 DNAzyme to promote the separation of ssDNA fragments after substrate cleavage in the presence of Pb^2+^. The isolated ssDNA adsorbed on AuNPs and prevented them from assembly at high ionic strength. As a result, Pb^2+^ caused a rapid color change of the AuNP solution from blue to red. The limit of detection of Pb^2+^ based on this design was as low as 0.2 nM.

#### 3.2.2. Detection by Modulating Electrostatic Interaction of DNA-Grafted AuNPs

In the above strategy, DNA can adsorb onto and desorb from AuNPs depending on its recognition behavior to heavy metal targets. The resultant interfacial electrostatic force among AuNPs is responsive for heavy metal detection. When DNAs are grafted on AuNP surfaces, the DNA-AuNPs need to be considered as a whole when analyzing the electrostatic interactions among them.

For instance, folded DNA exhibits high charge density compared to its unfolded counterpart, and the resultant stronger electrostatic repulsion between folded DNA are capable of stabilizing AuNPs against salt-induced assembly (Figure 3b) [70]. It has also been reported that the folding event of ssDNA densely grafted on AuNPs can cause the release of some of the DNA molecules from the AuNP surface [107]. By using the different strength of positively charged methylene blue (MB) to ssDNA and folded DNA, interestingly, Wang et al. developed a magnetoplasmonic nanoparticles (Fe_3_O_4_@AuNP)-based colorimetric sensor for detection of Hg^2+^ that is dependent on the color indication with MB [108]. As a positively charged organic dye with aromatic rings, MB binds more tightly to dsDNA than ssDNA by intercalating into dsDNA through π-π stacking [109], leading to a color change of the solution when the conformation of DNA grafted on Fe_3_O_4_@AuNP transformed from hybridization to folding. In the absence of Hg^2+^, aptamers on AuNPs hybridized with two ssDNA segments. The resulting long DNA polymers bound to MB, and thus, the solution color underwent a great decay upon magnetic separation. However, the recognition of Hg^2+^ with DNA-AuNPs on Fe_3_O_4_ resulted in transformation of the DNA derivative from a linear to a hairpin structure by forming T−Hg^II^−T, which has a lower binding capability to methylene blue. Therefore, a pronounced blue color was obtained upon magnetic separation. The absorbance of the residual MB solution was measured at 663 nm for quantification of Hg^2+^ with a LOD of 0.7 nM and a linear range of 1−300 nM. The above results also indicated the considerable potential of engineering multiple interfaces of DNA-AuNPs on single Fe_3_O_4_ nanoparticles.

### 3.3. Base Pair Stacking-Mediated Assembly for Detection

**Figure 4:**
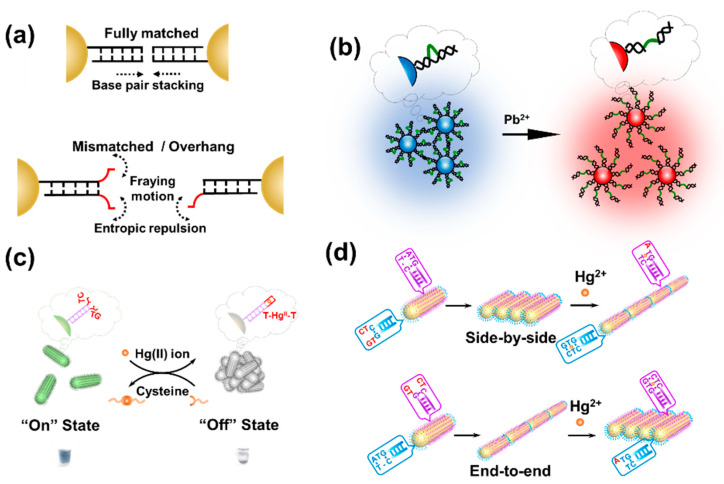
Base pair stacking-mediated assembly of DNA-AuNPs for heavy metal detection. (**a**) Schematic illustration of the entropic force among AuNPs controlled by base paring of DNA terminals. (**b**) The Pb^2+^ detection system based on the decreased rigidity of the DNAzyme grafted on the surface of AuNP. (**c**) Switching of dsDNA-AuNRs’ assembly state regulated by Hg^2+^/Cysteine-controlled DNA terminal base paring. (**d**) Schematic diagrams for the Hg^2+^-directed assembly of AuNRs achieved by regioselective modification of DNA with fine designed terminals.

Different from the above strategies, the control of single base pairing/unpairing in the outermost terminals of DNA that grafted on AuNPs has recently been developed for visual detection. According to the pioneering works of Maeda and co-workers, for AuNPs that are densely modified with dsDNA, the pairing status of the outermost base(s) of the dsDNA-AuNPs do matter to the colloidal stability. When DNA terminal bases are complementary, there is hydrophobic interaction (e.g., π-π stacking) between the blunt ends, which causes a sharp decay in the colloid stability of AuNPs and thereby rapid assembly in a non-crosslinking fashion (Figure 4a) [72,73,75].

When the terminal bases are mismatched, the AuNPs remain steadily dispersed even at a high salt concentration. This can be attributed to the fraying motion of the unpaired bases that generates strong entropic repulsion. Since the outermost base pairs can be designated to chelate heavy metals, such as Hg^2+^ and Ag^+^, the non-crosslinking assembly of DNA-AuNPs have implications in heavy metal detection. Kanayama et al. designed a nano-sensor that can visually detect Hg^2+^ in 1 min [77]. On account of the T−T mismatch at the antepenultimate position of DNA, the dsDNA-AuNP colloid remains highly stable and dispersed. By adding Hg^2+^ to form a T−Hg^II^−T complex, the entropic repulsion decreased and the AuNPs aggregated and precipitated rapidly, resulting in a distinct color change from red to colorless. The visual detection limit of this method for Hg^2+^ was estimated to be ~0.5 µM. By fine design of the terminal bases of DNA grafted on AuNPs, they further constructed a series of colloidal logic gates regulated by T−Hg^II^−T and C−Ag^I^−C formation [76]. The rigidity of dsDNA can also be modulated to control colloidal entropic repulsion. Diao et al. constructed a “turn-off” detection system for Pb^2+^ with GR-5 DNAzyme-grafted AuNPs [110], which have blunt ends at the outermost terminal of the DNAzyme (Figure 4b). Therefore, the AuNPs aggregated as a result of stacking attraction. In presence of Pb^2+^, the substrate chain would be cleaved, and the loop of the enzyme chain became flexible and greatly enhanced the colloidal entropic repulsion., bringing the AuNP aggregates back to the state of dispersion. The detection limit of this method was 8.0 nM, which was lower than that reported for the electrochemical detection method [111].

In addition to spherical nanoparticles, the entropic interfacial behavior is also applicable to the nanostructures with different morphologies, such as gold nanorod (AuNR) and gold nanotriangle (AuNT) [38]. Zhang et al. prepared a plasmon switch to rapidly detect two targets based on dsDNA-AuNRs that show a T−T mismatch at the penultimate position (Figure 4c) [112]. In the presence of Hg^2+^, the T−T mismatch of the dsDNA-AuNRs are transformed into a T−Hg^II^−T pairing, causing spontaneous non-crosslinking assembly of the dsDNA-AuNRs. On the contrary, the resultant dsDNA−AuNRs that have T−Hg^II^−T at the outermost dsDNA layer have Hg^2+^ extracted to reproduce the T−T mismatch upon introduction of cysteine, causing the color shift from colorless back to green. This switch can be quickly and repeatedly cycled at room temperature, and be used to detect low-concentration Hg^2+^ down to ~10 nM. This concentration reaches the maximum allowable level of Hg^2+^ in drinking water as defined by EPA [113].

One unique characteristic of anisotropic nanoparticles is the uneven surface reactivity. For instance, the ends of AuNR are more active in ligand attachment and metal deposition due to the large curvature and less stable crystal structure [114,115]. By region-selective DNA modification, Wang et al. achieved directed assembly of AuNRs in side-by-side and end-to-end manners (Figure 4d) [48]. By further anchoring specifically designed Hg^2+^-binding DNA on the ends or side region of the AuNRs, it is possible to prepare Hg^2+^-responsive side-by-side and end-to-end assemblies of AuNRs.

In comparison to its crosslinking counterpart, the non-crosslinking assembly of AuNPs has the advantage of rapid color response in the presence of an excess amount of target [67,116], thereby promising gene diagnostics once combined with polymerase chain reaction (PCR) [117]. Low-polarity solvents also facilitate the entropic interfacial assembly of DNA-AuNPs, making it suitable for heavy metal detection in expanded contexts [118].

## 4. Comparison among the Three Strategies for Heavy Metal Detection Based on DNA-AuNPs

By using the approaches based on electrostatic interactions, crosslinking or base pair stacking, the change of assembly state of the DNA-AuNPs caused by the recognition behavior of the functional DNAs generates the colorimetric signal for target heavy metals. However, the three approaches have their own advantages and disadvantages. Representative DNA-AuNP probes designed using the three strategies are listed in Table 1, and the selectivity of each methods were evaluated by the signal intensities of interfering ions. If the signals of more than one interfering ion are much higher than blank, the selectivity were judged to be moderate. According to the literature discussed above, the strategy of electrostatic interaction-mediated assembly based on DNA adsorption onto AuNPs is relatively easy to design and widely employed for heavy metal sensing due to the target-responsive structural switching feature of ssDNA, which often causes a change in the charge distribution of DNA. However, it is associated with the nonspecific adsorption of DNA on AuNPs, often leading to broad distribution of equilibrium constant in desorption [119]. Both the crosslinking- and entropic force-based methods, which mostly rely on the use of DNA-grafted AuNPs, avoid the problem of non-specific adsorption, but they are limited to the detection of Hg^2+^ and Pb^2+^ despite improved selectivity. An advantage of base pair stacking-based method is the capability of detection at high ionic strength, which promises heavy metal detection in real samples that are probably difficulty to achieve with the other two methods.

## 5. Conclusions and Perspectives

Since the discovery of functional DNAs for metals including aptamers and DNAzyme, detection of heavy metals based on the combination of DNA and AuNPs has been a lasting research interest in the relevant fields. With hundreds of papers published yearly, great progress has been achieved in the field of DNA-AuNPs for heavy metal sensing. For instance, many detection approaches based on the DNA-binding responsive interface of DNA-AuNPs have been developed, such as electrostatic interactions, crosslinking and base pair stacking. Additionally, the LOD for their colorimetric detection has also been lowered by many orders of magnitude. Thanks to the tremendous efforts people have devoted to this research field, recent years have also witnessed a quiet shift of research interest in DNA-AuNPs-based biosensing from principle-of-concept demonstration in the laboratory to practical applications for real samples. Portable devices, such as test strips, have gained an increasing popularity for field analysis with a computer or a smartphones as a data processor [120,121]. Regarding applications in fields ranging from environmental and biological analysis to food safety, challenges and opportunities are ahead as well. First, a major intrinsic problem in heavy metal detection using interface-engineered DNA-AuNPs is the relative low affinity in metal binding, particularly in the nanomolar range. This may cause a weak response of the DNA-AuNP interface to a heavy metal target, resulting in low detection sensitivity and selectivity. It is, thus, suggested that the selection of aptamers for heavy metals with higher affinity is urgently needed. Second, the binding mechanism between an aptamer and its heavy metal target (e.g., Cd, As) mostly remain less known, which is essential for finer design of interface-driven DNA-AuNP probes. Third, although detection of the typical heavy metal ions including Hg^2+^, Pb^2+^, Cd^2+^ and As^3+^ has received remarkable progress, in nature the heavy metals exist in a range of oxidation states as organic compounds, which exhibit high toxicity to human health and the ecological system. Accordingly, the real-life application of DNA-AuNPs for environmental samples is urgently needed. Finally, the exploitation of smart optical readers, including smartphones, would be a future direction for biosensors based on DNA-AuNPs. Based on the integration of gold nanostructures as indicators and the portable color readers for quantification, convenient and multiplex colorimetric detection of heavy metals for in-field analysis can be expected.

## Figures and Tables

**Table 1:** Colorimetric detection of heavy metals by different strategies based on the use of DNA-AuNPs.

Mechanisms	Targets	LOD	Selectivity	Ref.
Crosslinking	Hg^2+^/CH_3_Hg^+^	15/1.7 µM	Good	[83]
	Hg^2+^	100 nM	Moderate	[84]
	Hg^2+^	3 µM	Good	[85]
	Pb^2+^	100 nM	Moderate	[89]
	Pb^2+^	400 nM	Good	[90]
	Pb^2+^	0.05 nM	Good	[91]
Electrostatic interaction	Hg^2+^	250 nM	Good	[95]
	Hg^2+^	50 nM	Moderate	[97]
	As^3+^	16.8 nM	Moderate	[99]
	Arsenite	4.0 nM	Moderate	[101]
	Cd^2+^	4.6 nM	Moderate	[103]
	Pb^2+^	3 nM	Good	[105]
	Pb^2+^	0.2 nM	Moderate	[106]
	Hg^2+^	0.7 nM	Moderate	[108]
Base pair stacking	Hg^2+^	0.5 µM	Good	[77]
	Hg^2+^	10 nM	Good	[112]
	Pb^2+^	8.0 nM	Moderate	[110]
